# Multimodal Analgesia and Outcomes in Hysterectomy Surgery—A Population-Based Analysis

**DOI:** 10.3390/jcm13185431

**Published:** 2024-09-13

**Authors:** Crispiana Cozowicz, Hannah D. Gerner, Haoyan Zhong, Alex Illescas, Lisa Reisinger, Jashvant Poeran, Jiabin Liu, Stavros G. Memtsoudis

**Affiliations:** 1 Department of Anesthesiology, Perioperative Medicine and Intensive Care Medicine, Paracelsus Medical University, Muellner Hauptstrasse 48, 5020 Salzburg, Austria; c@cozowicz.at; 2 Medical University of Graz, Neue Stiftingtalstrasse 6, 8010 Graz, Austria; hannah.gerner@stud.medunigraz.at; 3 Department of Anesthesiology, Critical Care & Pain Management, Weill Cornell Medical College, Hospital for Special Surgery, 535 East 70th Street, New York, NY 10021, USA; zhongh@hss.edu (H.Z.); illescasa@hss.edu (A.I.); reisingerl@hss.edu (L.R.); poeranv@hss.edu (J.P.); liuji@hss.edu (J.L.); 4 Department of Anesthesiology, Weill Cornell Medicine, New York, NY 10021, USA

**Keywords:** hysterectomy, multimodal analgesia, anesthesia, pain management, perioperative, complications, opioids

## Abstract

**Objective:** We aimed to investigate the impact of multimodal analgesia on postoperative complications and opioid prescription on a national level. **Methods**: This retrospective cross-sectional study included n = 1,307,923 hysterectomies (01/2006–12/2022, Premier Healthcare claims data). Multimodal analgesia was defined as opioid use with the addition of non-opioid analgesic modes, grouped into four categories: opioid-only and 1, 2, or 3 or more additional non-opioid analgesics. Multivariable regression models measured associations between multimodal categories and outcomes (composite/respiratory/cardiac/gastrointestinal/genitourinary, and CNS complications, oral morphine milligram equivalents [MME], and length of hospital stay [LOS]). Odds ratios (OR) and 95% confidence intervals (CI) are reported. **Results**: Overall, 84.3% (1,102,812/1,307,923) received multimodal analgesia, of which 58.9%, 28.0%, and 13.1% received 1, 2, or 3 or more additional non-opioid analgesics, respectively. The odds of any composite complication (any ≥1 complication) decreased with the addition of 1, 2, 3, or more analgesic modalities (versus opioid-only): OR 0.66 (CI 0.64; 0.68), OR 0.63 (CI 0.61; 0.66), OR 0.65 (CI 0.62; 0.67), respectively. Similar patterns existed for respiratory, cardiac, and genitourinary complications. Opioid prescription decreased incrementally with 1,2, 3, or more non-opioid analgesic modalities by 9.51 mg (CI 11.16; 7.86) and 15.29 mg (CI 17.21; 13.37) and 29.35 mg (CI 31.79; 26.91) cumulative MME. LOS was reduced by 0.52 days (CI 0.54; 0.51), 0.49 days (CI 0.51; 0.47), and 0.40 days (CI 0.43; 0.38), respectively. Costs were reduced by $765 (CI 817; 714) or $479 (CI 539; 419) with 1 or 2 multimodal modes. **Conclusions**: These findings suggest substantial benefits of multimodal analgesia, including significant decreases in serious complications (especially respiratory, cardiac, and genitourinary), opioid consumption, and hospitalizations. Multimodal analgesia may facilitate safe and efficient pain management with optimized opioid consumption.

## 1. Introduction

In the United States, hysterectomies are the most frequent non-obstetric surgical procedures performed in women, rendering an incidence of more than 600,000 per year [1]. Regardless of surgical route, postoperative pain is significant and may escalate to chronic conditions, as reported in 10–50% [2]. While insufficient pain management presents a driver of delayed recovery and complications, the prevalent opioid crisis has raised concern for excessive perioperative opioid consumption. Thus, efforts to optimize perioperative care and accelerate patient recovery have spurred the implementation of enhanced recovery after surgery (ERAS) pathways with multimodal analgesic techniques as key interventions for improved postoperative outcomes [3]. Multimodal analgesia concurrently targets different pain pathways to facilitate additive and synergistic analgesic effects [4]. The aim is to attenuate the surgical stress response, reduce opioid consumption, and improve patient recovery. While a growing body of evidence recommends the utilization of multimodal analgesia, little is known about nationwide utilization practices and added clinical benefits in hysterectomy surgery. Moreover, population-based data on the impact of multimodal analgesia in terms of postoperative complication risk, patient recovery, and opioid consumption are lacking.

We therefore utilized national data to investigate associations between the use of multimodal analgesia and the occurrence of postoperative complications, length of hospital stay (LOS), and opioid prescription in patients undergoing hysterectomy surgery. We hypothesized that multimodal analgesia would be associated with a reduction in postoperative complications, LOS, and opioid use.

## 2. Methods

### 2.1. Study Design and Sample

This retrospective cross-sectional study was approved by the Institutional Review Board (IRB# 2012-050) of the Hospital for Special Surgery, New York. The requirement for written informed consent was waived, given the de-identified nature of these data. We identified adult patients undergoing elective hysterectomy surgery (2006–2022) from the Premier Healthcare claims dataset. (Premier Healthcare Solutions, Inc., Charlotte, NC, USA). Hysterectomy procedures were identified utilizing international classification of diseases -ninth/tenth revision (ICD-9/10 codes) and The Current Procedural Terminology (CPT) codes as shown in Appendix A. Exclusion criteria encompassed unknown sex (n = 516), unknown discharge status (n = 1533), patients without documented opioid use (n = 70,635), and cases with opioid utilization above the 95th percentile (to address outliers, n = 13,355).

### 2.2. Study Variables

The exposure of interest was the implementation of multimodal analgesia, defined as opioid use with the addition of 1, 2, or >2 non-opioid analgesic modes, as previously operationalized. Non-opioid analgesic modes included ketamine, non-steroidal anti-inflammatory drugs (NSAIDs), cyclooxygenase-2 inhibitors, paracetamol/acetaminophen, steroids (>1 dose of use on the day of surgery or the day after surgery), gabapentin/pregabalin, or neuraxial anesthesia. The use of multimodal analgesia components was identified from billing codes, which were published by this study group previously [4,5].

The outcomes of interest were perioperative complications (see below), opioid prescription, LOS measured in days, and cost measured in 2022 United States dollars (USD). Complications included a composite (any ≥1 complication) and individual analysis of respiratory, cardiac, gastrointestinal, genitourinary, and central nervous system (CNS) complications. All complications were defined using ICD-9 and ICD-10 diagnosis codes based on the previous literature (see Appendix B for definitions). Opioid utilization during hospitalization was based on billing for opioids (opioid type and dosage were billed by day in Premier) and then was converted into oral morphine milligram equivalents (MME) [6].

Patient-level variables included age, sex, race (black, white, other), insurance type (commercial, Medicaid, Medicare, uninsured, other), Elixhauser Comorbidity index (categorized as 0, 1, 2, 3+), history of sleep apnea, substance abuse, chronic pain, psychiatric, and opioid abuse. Procedure-level variables included the year of the procedure and type of anesthesia (general, neuraxial). Healthcare-level variables included hospital location (urban, rural), hospital size (<300, 300–499, ≥500 beds), and hospital teaching status.

### 2.3. Statistical Analysis

A descriptive analysis of all study variables was presented and stratified by the number of multimodal analgesia modalities used. Categorical variables were presented as counts and percentages, and continuous variables were presented as median and interquartile ranges (IQR). Given our large sample size, univariable differences between groups easily reach statistical significance; therefore, we applied standardized differences instead of *p*-values. An absolute standardized difference of ≥0.1 (or 10%) generally indicates a meaningful difference in covariate distribution between groups [7].

Multivariable multilevel logistic regression models were run to compare associations between the use of multimodal analgesia categories for all binary outcomes (composite complication and individual complications). Odds ratios (OR) and 95% confidence intervals (CI) were reported. The models were adjusted for all priori-determined covariates, including age, race, gender, Elixhauser Comorbidity index group, insurance type, year of surgery, hospital location, bed size, and hospital teaching status, type of anesthesia, history of sleep apnea, substance abuse, chronic pain, psychiatric history, and opioid abuse, based in their potential association with exposure and outcome, as previously described [5,8]. A generalized linear model was applied to compare associations between the use of multimodal analgesia categories (categorized as opioid-only, 1, 2, or >2 non-opioid analgesic modes) and continuous outcomes (opioid utilization, LOS, and cost), adjusting the same covariates as in the logistic regression model. Estimates were presented as least square means differences compared with the reference group along with 95% CIs. For the opioid utilization outcome, we additionally adjusted for substance use/abuse (including smoking), chronic pain conditions, psychiatric comorbidity variables, and opioid use disorder, given their association with opioid use. The outcome of LOS was modeled using only the inpatient cohort. A *p*-value <0.01 was used as the cutoff for statistical significance. Analyses were performed with SAS version 9.4 (SAS Institute, Cary, NC, USA).

## 3. Results

Among 1,307,923 adult women undergoing hysterectomy surgery from January 2006 to December 2022, we found that 84.3% (n = 1,102,812) received multimodal analgesia, of which 58.9%, 28.0%, and 13.1% received 1, 2 or >2 additional non-opioid analgesics, respectively. The use of >2 analgesic techniques for multimodal analgesia (compared with those on opioids only) was most common in teaching hospitals. In contrast, in non-teaching hospitals, a lower number of analgesic modalities were utilized more frequently. Multimodal analgesia utilization was slightly higher among those with an increased comorbidity burden and a history of substance abuse, while lower use was seen for older patients, those on Medicare, and patients receiving general anesthesia; all standardized differences ≥0.1 (Table 1).

**Figure 1:**
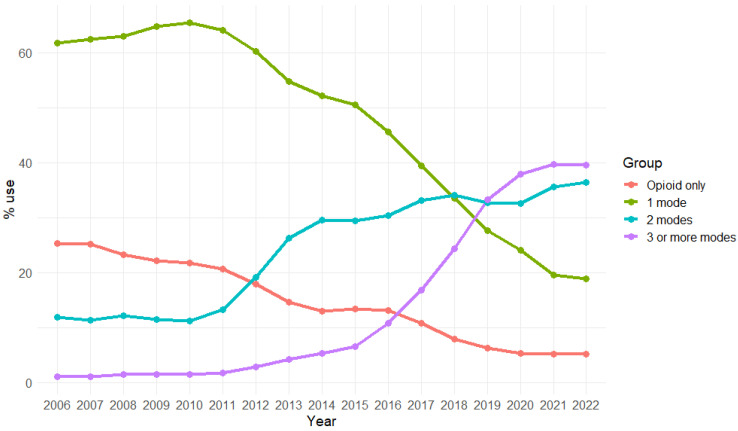
Trends of multimodal analgesia use among hysterotomy cases over study time.

The trend analysis showed that the practice of adding three or more non-opioid analgesic modalities to opioids has seen a steady rise from an incidence of 1.03% in 2006 to 39.57% in 2022. Concurrently, the use of just one non-opioid analgesic modality in addition to opioid analgesia decreased from 61.72% in 2006 to 18.84% in 2022. Furthermore, a drop in the use of opioid-only-based analgesia was observed from 25.31% in 2006 to 5.14% in 2022 (Figure 1). The most frequently used non-opioid analgesics were NSAIDs and acetaminophen, followed by gabapentin and neuraxial anesthesia (Table 1).

In unadjusted outcome comparisons, patients receiving multimodal analgesia had a lower incidence of the composite of any complication and, more specifically, respiratory, cardiac, and genitourinary complications. Furthermore, opioid dispensation was lower among those receiving multimodal analgesia (Table 2).

This pattern was more pronounced in adjusted models. (Table 3) Multimodal use applied as a categorical variable with four categories (1, 2, or 3 or more additional non-opioid analgesics versus opioid-only) was associated with consistent decreases in any complication (OR 0.66 CI 0.64; 0.68/OR 0.63 CI 0.61; 0.66/OR 0.65 CI 0.62; 0.67). The increased modes of analgesia did not result in more reduced odds of complications. Consistently, respiratory complications decreased by (OR 0.60 CI 0.57; 0.63/OR 0.60 CI 0.56; 0.64/OR 0.64 CI 0.60; 0.69), cardiac complications by (OR 0.71 CI 0.68, 0.75/OR 0.69 CI 0.65, 0.73/OR 0.66 CI 0.62, 0.71), and genitourinary complications decreased by (OR 0.65 CI 0.62, 0.68/OR 0.58 CI 0.55, 0.61/OR 0.61 CI 0.57, 0.66) with the addition of 1,2, or 3 or more multimodal techniques, respectively; all *p* < 0.001. A decrease in gastrointestinal complications was only observed with the addition of one non-opioid analgesic modality OR 0.81 (CI 0.74, 0.90). No effect was found for the occurrence of CNS system complications.

Notably, opioid prescription was incrementally reduced in line with additional non-opioid analgesics utilized: −9.51 mg (CI −11.16; −7.86) and −15.29 mg (CI −17.21; −13.37) and −29.35 mg (CI −31.79; −26.91). LOS decreased by 0.52 days (CI 0.54; 0.51), 0.49 days (CI 0.51; 0.47), and 0.40 days (CI 0.43; 0.38), respectively. Furthermore, cost reductions of −765 USD (CI −817; −714) and −479 USD (CI −539; −419) were observed with 1 or 2 modalities. However, a small, non-significant increase in the cost of 79 USD (CI 2; 156) emerged among those receiving >2 non-opioid analgesic modalities (Table 3).

## 4. Discussion

In this large national sample of more than 1.3 million women undergoing hysterectomy surgery from 2006 to 2022, we found that the use of multimodal analgesia was associated with significantly improved outcomes.

The odds of the occurrence of any postoperative complication were reduced by 35%. More specifically, respiratory complications decreased by about 40%, cardiac complications by 30%, and genitourinary complications were reduced by 40% when compared with patients without multimodal analgesia. No impact on the occurrence of CNS complications was observed. Concurrently, opioid prescription incrementally decreased with the use of increasing modes of multimodal modalities. Furthermore, we observed a reduction in LOS while costs were reduced with the addition of 1 or 2 analgesic modalities, but not when adding >2 analgesic techniques.

Trend analysis demonstrated substantial changes in the practice of perioperative pain management for hysterectomies in the last two decades. Most notably, a steep increase in the use of >2 non-opioid analgesic modalities was found, which increased from an incidence of 1.03% in 2006 to 39.57% in 2022. Concurrently, the use of just one non-opioid analgesic modality in addition to opioid analgesia decreased from 61.72% in 2006 to 18.84% in 2022.

Hysterectomy is the second most common procedure performed in women after obstetric surgery, which is in part related to gynecological malignancy affecting over 1,000,000 women per year [3,9]. Although it is known that hysterectomies often result in significant pain and slow recovery, postoperative pain is easily overlooked, and persistent opioid use is reported in 5% regardless of surgical route [9,10,11]. Inadequate analgesic management after gynecologic surgery is a major driver of postoperative complications, delayed recovery, and increased opioid use [12,13].

Therefore, enhanced recovery after surgery pathways have been widely implemented, including the gynecological specialties, with strong recommendations for the routine administration of multimodal analgesia. The goal is to concurrently target various pain pathways by combining different analgesic medications and techniques to achieve additive and synergistic analgesic effects while diminishing complications [12]. However, little is known about the clinical impact of multimodal analgesia in hysterectomies, and pharmacologic strategies to improve the quality of this population have yet to be examined [14]. In this context, these data demonstrate that in a large sample of hysterectomies, the use of multimodal analgesia was associated with a substantial decrease in the occurrence of serious adverse events, including respiratory, cardiac, and genitourinary complications. Pain physiologically confers several detrimental effects, such as adrenergic activation with catecholamine release, inflammatory mediator activation, hemodynamic instability, cardiac stress, and respiratory deterioration [5]. Thus, inadequate analgesia is linked to increased perioperative morbidity with a higher risk for cardiac and pulmonary complications [15,16,17,18,19]. It is, therefore, conceivable that multimodal analgesia, by concurrently targeting multiple pain pathways, may diminish postoperative stress and other pain-related detriments and, therefore, accelerate postoperative recovery. Consistently, these data also demonstrate a decrease in LOS among patients with multimodal analgesia. This is in line with evidence from radical laparoscopic gynecological cancer surgery, where multimodal analgesia conferred lower pain scores, earlier mobilization, and a lower incidence of severe complications [13].

While we did not have information on pain scores in these data, we found that multimodal analgesia was associated with a significant reduction in opioid prescription, which could indicate improved pain management [10]. With the opioid crisis globally expanding, the pressure to restrict or even abolish opioid use has increasingly gained popularity in clinical practice. At the same time, postoperative pain remains insufficiently managed, with reported incidences reaching 80% [20]. This is a serious concern given that pain appears to be the most prevalent, disabling, and burdensome health problem in the US, with related expenses exceeding those for cardiac disease, cancer, and diabetes. [20,21]. Therefore, perioperative clinicians are particularly challenged with facilitating adequate pain management and rapid recovery while trying to diminish drivers of persistent opioid use. In the absence of equipotent alternatives, opioids remain the mainstay for perioperative pain management based on their undisputed analgesic efficacy [20,22]. It may, therefore, be more meaningful to strive for improved postoperative pain management and fastened recovery rather than exclusive opioid reduction [20]. In fact, opioid-free strategies have not been shown to decrease the risk of persistent opioid use [23]. The complexity in hysterectomies is highlighted by evidence showing that hysterectomy surgeons appear to be among the top physician prescribers of opioids nationally, while in laparoscopic hysterectomies, it has been shown that opioid prescriptions were a four-fold of what was required for acute postoperative pain control [24,25]. Moreover, nationwide trend analysis showed a substantial increase in perioperative opioid prescriptions for hysterectomies from 2004 to 2014 despite an increase in minimally invasive surgical techniques [26]. In this national sample of hysterectomies, we observed an incremental decrease in opioid prescription with the use of increasing modes of multimodal analgesia techniques. This dose–response relationship was observed alongside a reduced risk for serious postoperative complications and a significant decrease in LOS. Consistently, opioid-sparing with multiple analgesic modalities has been shown in laparoscopic hysterectomies and cesarean deliveries [10]. Previously, however, no conclusions could have been drawn regarding patient safety and complication risk due to the lack of adequately powered data [27]. The current data supports the notion of improved postoperative outcomes with reduced opioid consumption in hysterectomy surgery. With the Society of Gynecologic Oncology encouraging limited opioid use, multimodal analgesia may prove to be crucial in the development of perioperative strategies that strike a balance between adequate pain control and the prevention of excess opioid use [9,28].

## 5. Limitations

This analysis is subject to several limitations, given the nature of observational data. Despite efforts to account for baseline differences, the lack of randomization bears the risk of residual confounding. Thus, causal conclusions cannot be drawn, while associations require careful interpretation in the context of plausibility based on previous scientific findings. As mentioned, patients without documentation for opioid use were excluded as reflective of either missing data or lack of opioid use. However, we would not anticipate a significant impact on our results as missing data would likely be evenly distributed, and the lack of opioid use would not comply with our predefined study question. Despite the nature of the surgery, patients with a lack of information on gender were excluded to eliminate any concerns about quality issues in these cases. Furthermore, the lack of detailed clinical information (e.g., precise timing of medication and pain scores) needs to be acknowledged, which is based on the primary purpose of the utilized billing data. Analysis specific to individual ethnicities was not possible because of changes in reporting practices over the study period. Given the skewed distribution of age, LOS, cost, and opioid use, we reported median and IQR. Nevertheless, this is a large national sample of patients undergoing hysterectomy surgery, facilitating the investigation of potential harm.

## 6. Conclusions

This large national sample of patients undergoing hysterectomy surgery indicates substantial benefits with the use of multimodal analgesia. A decrease in serious complications by at least 30% was accompanied by incrementally reduced opioid use and a reduction in LOS. While more evidence is needed, multimodal analgesic approaches may facilitate safe and efficient pain management with optimized opioid consumption.

## Figures and Tables

**Table 1:** Baseline characteristics stratified by multimodal groups; each multimodal group is compared separately with the opioid-only group.

	Multimodal						
	Opioid-Only	1 Mode	Stdiff	2 Modes	Stdiff	3 or More Modes	Stdiff
	N = 200,052	N = 649,471		N = 308,579		N = 144,762	
**Age, median [IQR]**	48 [41.1, 59.9]	45.1 [39, 52.4]	−0.3	45 [38, 52.9]	−0.33	44.7 [36.9, 54.6]	−0.31
**Race, n (%)**							
Black	30,292 (15.1)	94,903 (14.5)	−0.02	46,790 (15.1)	<0.01	26,283 (18.1)	0.08
Other	39,651 (19.7)	114,842 (17.6)	−0.06	55,087 (17.8)	−0.05	22,948 (15.8)	−0.1
White	130,961 (65.2)	443,127 (67.9)	0.06	207,457 (67.1)	0.04	95,582 (66.0)	0.02
**Insurance, n (%)**			0.22		0.22		0.24
Commercial	124,528 (62.0)	451,696 (69.2)		202,136 (65.3)		86,796 (59.9)	
Medicaid	22,835 (11.4)	80,029 (12.3)		49,614 (16.0)		27,805 (19.2)	
Medicare	40,617 (20.2)	77,402 (11.9)		39,092 (12.6)		21,301 (14.7)	
Uninsured	5764 (2.9)	18,881 (2.9)		7999 (2.6)		4283 (3.0)	
Unknown	7160 (3.6)	24,864 (3.8)		10,493 (3.4)		4628 (3.2)	
**Inpatient/outpatient, n (%)**			−0.1		−0.1		0.06
Inpatient	143,079 (71.2)	433,476 (66.4)		205,441 (66.4)		107,018 (73.9)	
Outpatient	57,825 (28.8)	219,396 (33.6)		103,893 (33.6)		37,795 (26.1)	
**General anesthesia, n (%)**	169,508 (84.4)	549,005 (84.1)	−0.03	234,946 (76.0)	−0.33	95,309 (65.8)	−0.58
**Elixhauser comorbidity group, n (%)**			0.18		0.12		0.27
0	129,723 (64.6)	455,374 (69.7)		195,770 (63.3)		75,067 (51.8)	
1	32,755 (16.3)	109,647 (16.8)		60,494 (19.6)		33,106 (22.9)	
2	19,810 (9.9)	52,858 (8.1)		31,351 (10.1)		20,110 (13.9)	
3	18,616 (9.3)	34,993 (5.4)		21,719 (7.0)		16,530 (11.4)	
**Sleep apnea, n (%)**	4361 (2.2)	10,869 (1.7)	−0.04	6732 (2.2)	<.01	4646 (3.2)	0.06
**Substance abuse, n (%)**	24,323 (12.1)	86,390 (13.2)	0.04	44,193 (14.3)	0.06	26,159 (18.1)	0.17
**Chronic pain**	48,688 (24.2)	108,550 (16.6)	−0.19	52,110 (16.8)	−0.18	25,960 (17.9)	−0.16
**Psychiatric, n (%)**	24,741 (12.3)	82,118 (12.6)	0.01	39,573 (12.8)	0.01	18,658 (12.9)	0.02
**Opioid abuse, n (%)**	170 (0.1)	625 (0.1)	<0.01	562 (0.2)	0.03	574 (0.4)	0.06
**Urban/Rural, n (%)**			0.07		0.08		0.03
Rural	17,501 (8.7)	71,336 (10.9)		34,775 (11.2)		13,899 (9.6)	
Urban	183,403 (91.3)	581,536 (89.1)		274,559 (88.8)		130,914 (90.4)	
**Hospital Size, n (%)**							
Large	73,566 (36.6)	200,558 (30.7)	−0.13	109,264 (35.3)	−0.03	59,044 (40.8)	0.09
Medium	68,461 (34.1)	230,322 (35.3)	0.03	107,828 (34.9)	0.02	47,390 (32.7)	−0.03
Small	58,877 (29.3)	221,992 (34.0)	0.1	92,242 (29.8)	0.01	38,379 (26.5)	−0.06
**Teaching hospital, n (%)**			−0.05		0.1		0.32
No	114,276 (56.9)	386,814 (59.2)		159,842 (51.7)		59,751 (41.3)	
Yes	86,628 (43.1)	266,058 (40.8)		149,492 (48.3)		85,062 (58.7)	
**Use of multimodal components, n (%)**							
**Gabapentin**	-	5500 (0.8)		25,924 (8.4)		86,137 (59.5)	
**Steroid**	-	2835 (0.4)		8866 (2.9)		10,356 (7.2)	
**Ketamine**	-	3245 (0.5)		17,725 (5.7)		44,896 (31.0)	
**NSAID**	-	558,994 (85.6)		285,111 (92.2)		126,480 (87.3)	
**Cox2**	-	3456 (0.5)		9034 (2.9)		49,178 (34.0)	
**Acetaminophen**	-	72,041 (11.0)		233,878 (75.6)		133,243 (92.0)	
**Neuraxial**	-	5669 (0.9)		29,817 (9.6)		26,452 (18.3)	

Stdiff: Multimodal groups were compared with the opioid-only group. An absolute standardized difference of 0.1 (or 10%) generally indicates a meaningful difference in covariate distribution between groups.

**Table 2:** Outcomes stratified by multimodal groups.

	Opioid Only	1 Mode	2 Modes	3 or More Modes
	N = 200,052	N = 649,471	N = 308,579	N = 144,762
**Composite outcome for complication, n (%)**	8661 (4.3)	13,979 (2.1)	7694 (2.5)	4915 (3.4)
Respiratory	2998 (1.5)	4006 (0.6)	2330 (0.8)	1615 (1.1)
Gastrointestinal	626 (0.3)	1274 (0.2)	571 (0.2)	263 (0.2)
Genitourinary	3653 (1.8)	5446 (0.8)	2442 (0.8)	1374 (0.9)
Central nervous system	345 (0.2)	600 (0.1)	373 (0.1)	288 (0.2)
Cardiac	2489 (1.2)	4425 (0.7)	2948 (1.0)	2104 (1.5)
**Total opioid use, median [IQR]**	237 [124, 412]	226 [130, 385]	202 [114, 360]	173 [96, 315]
**Adjusted cost, median [IQR]**	9683 [6950, 13,494]	9033 [6758, 12,210]	9515 [7083, 13,041]	10,280 [7638, 14,485]
	**N = 143,079**	**N = 433,476**	**N = 205,441**	**N = 107,018**
**Length of stay for inpatient cohort, median [IQR]**	2, [2, 3]	2 [1, 3]	2 [2, 3]	2 [2, 3]

**Table 3:** Adjusted outcomes.

Binary Outcomes	Unadjusted Odds Ratio (95% CIs)	*p*-Value	Adjusted Odds Ratio (95% CIs) ^a^	*p*-Value
**Composite complications**				
1 mode vs. opioid only	0.49 (0.47, 0.5)	<0.001	0.66 (0.64, 0.68)	<0.001
2 modes vs. opioid only	0.57 (0.55, 0.58)	<0.001	0.63 (0.61, 0.66)	<0.001
3 modes vs. opioid only	0.78 (0.75, 0.81)	<0.001	0.65 (0.62, 0.67)	<0.001
**Respiratory complications**				
1 mode vs. opioid only	0.41 (0.39, 0.43)	<0.001	0.6 (0.57, 0.63)	<0.001
2 modes vs. opioid only	0.5 (0.47, 0.53)	<0.001	0.6 (0.56, 0.64)	<0.001
3 modes vs. opioid only	0.74 (0.7, 0.79)	<0.001	0.64 (0.6, 0.69)	<0.001
**Cardiac complications**				
1 mode vs. opioid only	0.54 (0.52, 0.57)	<0.001	0.71 (0.68, 0.75)	<0.001
2 modes vs. opioid only	0.77 (0.73, 0.81)	<0.001	0.69 (0.65, 0.73)	<0.001
3 modes vs. opioid only	1.18 (1.11, 1.25)	<0.001	0.66 (0.62, 0.71)	<0.001
**Gastrointestinal complications**				
1 mode vs. opioid only	0.63 (0.57, 0.69)	<0.001	0.81 (0.74, 0.9)	<0.001
2 modes vs. opioid only	0.59 (0.53, 0.66)	<0.001	0.93 (0.83, 1.05)	0.242
3 modes vs. opioid only	0.58 (0.5, 0.67)	<0.001	1.08 (0.92, 1.28)	0.360
**Genitourinary complications**				
1 mode vs. opioid only	0.45 (0.44, 0.47)	<0.001	0.65 (0.62, 0.68)	<0.001
2 modes vs. opioid only	0.43 (0.41, 0.45)	<0.001	0.58 (0.55, 0.61)	<0.001
3 modes vs. opioid only	0.52 (0.49, 0.55)	<0.001	0.61 (0.57, 0.66)	<0.001
**Central nervous system complications**				
1 mode vs. opioid only	0.53 (0.47, 0.61)	<0.001	0.9 (0.79, 1.03)	0.143
2 modes vs. opioid only	0.7 (0.61, 0.81)	<0.001	0.93 (0.79, 1.09)	0.359
3 modes vs. opioid only	1.16 (0.99, 1.35)	0.066	0.99 (0.82, 1.19)	0.890
**Continuous Outcomes**	**Unadjusted least square means (95% CIs)**	***p*-value**	**Adjusted least square means (95% CIs) ^b^**	***p*-value**
**Length of stay for inpatients in days**				
1 mode vs. opioid only	−0.73 (−0.74, −0.71)	<0.001	−0.52 (−0.54, −0.51)	<0.001
2 modes vs. opioid only	−0.56 (−0.58, −0.54)	<0.001	−0.49 (−0.51, −0.47)	<0.001
3 modes vs. opioid only	−0.28 (−0.31, −0.30)	<0.001	−0.40 (−0.43, −0.38)	<0.001
**Adjusted cost in dollars**				
1 mode vs. opioid only	−1506 (−1560, −1452)	<0.001	−765 (−817, −714)	<0.001
2 modes vs. opioid only	−760 (−821, −700)	<0.001	−479 (−539, −419)	<0.001
3 modes vs. opioid only	666 (594, 739)	<0.001	79 (2, 156)	0.042
**Total opioid consumption**				
1 mode vs. opioid only	−16.55 (−18.24, −14.85)	<0.001	−9.51 (−11.16, −7.86)	<0.001
2 modes vs. opioid only	−32.34 (−34.25, −30.43)	<0.001	−15.29 (−17.21, −13.37)	<0.001
3 modes vs. opioid only	−63.57 (−65.86, −61.27)	<0.001	−29.35 (−31.79, −26.91)	<0.001

^a^. Multiple logistic regression models were run to compare associations between the use of multimodal analgesia categories for all binary outcomes (composite complication and individual complications). Odds ratios (OR) and 95% CIs were reported. Models were adjusted using a priori-determined covariate, including age, race, gender, Elixhauser Comorbidity index group, year of surgery, hospital location, bed size, and hospital teaching status, as described previously. ^b^. A generalized linear model was applied to compare associations between the use of multimodal analgesia categories (categorized as opioid-only, 1, 2, or >2 non-opioid analgesic modes) and continuous outcomes (Opioid utilization, inpatient length of stay, and cost) adjusting the same covariates in the logistic regression model. Estimates were presented as least square means differences compared with the reference group along with 95% CIs. For opioid utilization outcome, we additionally adjusted for a history of substance use/abuse (including smoking), chronic pain conditions, psychiatric comorbidity variables, and opioid use disorder, given their association with opioid use.

## Data Availability

Restrictions apply to the availability of these data. Data were obtained from Premier Healthcare and are available from Stavros G. Memtsoudis with the permission of Premier Healthcare.

## Appendix A

**Table A1:** Identifying hysterectomy procedures based on International Classification of Diseases 9th (ICD-9) and 10th Revision (ICD-10) procedure codes and The Current Procedural Terminology (CPT) codes.

ICD-9	Description
68.0x	Hysterotomy
68.3x	Subtotal abdominal hysterectomy
68.4x	Total abdominal hysterectomy
68.5x	Vaginal hysterectomy
68.6x	Radical abdominal hysterectomy
68.7x	Radical vaginal hysterectomy
68.8x	Pelvic evisceration
68.9x	Other and unspecified hysterectomy
**ICD-10**	
0U54xxx	Destruction of Uterine Supporting Structure
0U55xxx	Destruction of Fallopian Tube, Right
0U56xxx	Destruction of Fallopian Tube, Left
0U57xxx	Destruction of Fallopian Tubes, Bilateral
0U59xxx	Destruction of Uterus
0U94xxx	Drainage of Uterine Supporting Structure
0U95xxx	Drainage of Fallopian Tube, Right
0U96xxx	Drainage of Fallopian Tube, Left
0U97xxx	Drainage of Fallopian Tubes, Bilateral
0U99xxx	Drainage of Uterus
0UB4xxx	Excision of Uterine Supporting Structure
0UB5xxx	Excision of Fallopian Tube, Right
0UB6xxx	Excision of Fallopian Tube, Left
0UB7xxx	Excision of Fallopian Tubes, Bilateral
0UB9xxx	Excision of Uterus
0UC4xxx	Extirpation of Uterine Supporting Structure
0UC5xxx	Extirpation of Fallopian Tube, Right
0UC6xxx	Extirpation of Fallopian Tube, Left
0UC7xxx	Extirpation of Fallopian Tubes, Bilateral
0UN4xxx	Release of Uterine Supporting Structure
0UN5xxx	Release of Fallopian Tube, Right
0UN6xxx	Release of Fallopian Tube, Left
0UN7xxx	Release of Fallopian Tubes, Bilateral
0UN9xxx	Release Uterus
0UT2xxx	Resection of Bilateral Ovaries
0UT4xxx	Resection of Uterine Supporting Structure
0UT5xxx	Resection of Fallopian Tube, Right
0UT6xxx	Resection of Fallopian Tube, Left
0UT7xxx	Resection of Fallopian Tubes, Bilateral
0UT9xxx	Resection of Uterus
0UTCxxx	Resection of Cervix
**CPT**	
58150	Total abdominal hysterectomy (corpus and cervix), with or without removal of tube(s), with or without removal of ovary(s)
58152	Total abdominal hysterectomy (corpus and cervix), with or without removal of tube(s), with or without removal of ovary(s)
58180	The provider removes the uterus via an abdominal incision. The provider may also remove the fallopian tubes and ovaries.
58200	The provider removes the uterus and cervix and may also remove the fallopian tubes and ovaries, all through via an abdominal incision. The provider removes the upper one–third of the vaginal canal and some of the pelvic and para-aortic lymph nodes.
58210	The provider removes the uterus and cervix, including the parametrium, via an abdominal incision, known as a radical abdominal hysterectomy. The provider may also remove all or part of the vagina, all the pelvic lymph nodes on the right and left side, and biopsy a few of the para-aortic lymph nodes. The provider may remove part or all of the fallopian tubes and ovaries.
58240	The provider performs pelvic exenteration on patients who have had a recurrence of cancer of the cervix after they have had radiation therapy or patients who have stage IV cancer and the tumor is in the bladder and rectum. There is no standard procedure and the organs and tissues that the provider removes depend on where the cancer is located and the stage. In a total exenteration, the provider removes the uterus, tubes, ovaries, parametrial tissue, bladder, rectum, vagina, urethra, and part of the levator ani muscles. In an anterior exenteration, the provider does not remove the rectum. In a posterior exenteration, the provider does not remove the bladder and urethra. He may also resect part of the anus, urethra, and part of the vulva.
58260	In this procedure, the provider surgically removes the uterus and cervix only using a vaginal approach, known as a vaginal hysterectomy. The uterus is normal in size, which means it weighs 250 g or less.
58262	In this procedure, the provider surgically removes the uterus, cervix, fallopian tubes, and ovaries using a vaginal approach known as a vaginal hysterectomy. The uterus is normal in size, which means it weighs 250 g or less.
58263	In this procedure, the provider surgically removes the uterus, cervix, fallopian tubes, and ovaries using a vaginal approach known as a vaginal hysterectomy. Because the patient has a small bowel prolapsing into the vaginal canal, called an enterocele, he also repairs this area. The uterus is normal in size, which means it weighs 250 g or less.
58267	In this procedure, the provider surgically removes the uterus and cervix only using a vaginal approach, known as a vaginal hysterectomy. The patient also has stress urinary incontinence, which requires the suspension of the urethra. The uterus is normal in size, which means it weighs 250 g or less, and the provider may use an endoscope during the procedure.
58270	In this procedure, the provider surgically removes the uterus and cervix only using a vaginal approach, known as a vaginal hysterectomy. The provider also repairs a small bowel prolapse into the vaginal canal. The uterus is normal in size, which means it weighs 250 g or less.
58275	In this procedure, the provider surgically removes the uterus and the cervix using a vaginal approach, known as a vaginal hysterectomy. The provider also partially or completely excises the vagina.
58280	The provider surgically removes the uterus and the cervix using a vaginal approach, known as a vaginal hysterectomy. The provider also partially or completely excises the vagina and repairs a small bowel prolapse into the vaginal canal.
58285	the provider removes the uterus, fallopian tubes, and ovaries, the parametrium, which includes the uterosacral, cardinal, broad, and round ligaments, and the upper one-third of the vagina. This procedure treats cervical cancer.
58290	the physician surgically removes the uterus and cervix only using a vaginal approach. The uterus is larger than normal, usually due to the presence of fibroids, which means it weighs more than 250 g.
58291	the physician surgically removes the uterus, cervix, the fallopian tubes, and ovaries using a vaginal approach. The uterus is usually larger than normal because of the presence of fibroids, which means it weighs more than 250 g.
58292	the physician surgically removes the uterus, cervix, fallopian tubes, and ovaries using a vaginal approach. Because the patient has small bowel prolapse into the vaginal canal, the provider also repairs this area. The uterus is usually larger than normal due to the presence of fibroids, which means it weighs more than 250 g.
58294	the physician surgically removes the uterus and cervix only using a vaginal approach. Because the patient has a small bowel prolapsing into the vaginal canal, this area is repaired. The uterus is usually larger than normal because of the presence of fibroids, which means it weighs more than 250 g.

## Appendix B

**Table A2:** Complication identification based on the International Classification of Diseases, Ninth and Tenth Revision (ICD-9/ICD-10).

	ICD-9	ICD-10
Respiratory	518.5, 518.52, 518.51, 518.53, 518.82, 786.09, 799.02, 799.01, 518.81, 518.84	J95.2, J80, R09.02, R09.01, J95.821, J96.00, J96.01, J96.02, J96.90, J96.91, J96.92, J96.20, J96.21, J96.22, J95.822
Cardiac		
Gastrointestinal	584, N17	E879.1, V45.11, Z99.2
Genitourinary	997.4	K91.3, K91.8
CNS	997.01,780.09,780.97,799.2, 293	G97.81, G97.82, R41.82, R40.0, R40.1, F06.8, R45, F05, F6, F53

## References

[1] Harvey S.V., Pfeiffer R.M., Landy R., Wentzensen N., Clarke M.A. (2022). Trends and predictors of hysterectomy prevalence among women in the United States. Am. J. Obstet. Gynecol..

[2] Brandsborg B., Nikolajsen L. (2018). Chronic pain after hysterectomy. Curr. Opin. Anaesthesiol..

[3] Bisch S.P., Wells T., Gramlich L., Faris P., Wang X., Tran D.T., Thanh N.X., Glaze S., Chu P., Ghatage P. (2018). Enhanced recovery after surgery (eras) in gynecologic oncology: System-wide implementation and audit leads to improved value and patient outcomes. Gynecol. Oncol..

[4] Memtsoudis S.G., Poeran J., Zubizarreta N., Cozowicz C., Morwald E.E., Mariano E.R., Mazumdar M. (2018). Association of multimodal pain management strategies with perioperative outcomes and resource utilization: A population-based study. Anesthesiology.

[5] Cozowicz C., Zhong H., Poeran J., Illescas A., Liu J., Poultsides L.A., Avgerinos D.V., Memtsoudis S.G. The impact of multimodal analgesia in coronary artery bypass graft surgery-a population-based analysis. J. Thorac. Cardiovasc. Surg..

[6] Dowell D., Ragan K.R., Jones C.M., Baldwin G.T., Chou R. (2022). Cdc clinical practice guideline for prescribing opioids for pain—United States, 2022. MMWR Recomm. Rep..

[7] Austin P.C. (2011). An introduction to propensity score methods for reducing the effects of confounding in observational studies. Multivar. Behav. Res..

[8] Liu H., Zhong H., Zubizarreta N., Cagle P., Liu J., Poeran J., Memtsoudis S.G. (2024). Multimodal pain management and postoperative outcomes in inpatient and outpatient shoulder arthroplasties: A population-based study. Reg. Anesth. Pain. Med..

[9] Hessami K., Welch J., Frost A., AlAshqar A., Arian S.E., Gough E., Borahay M.A. (2023). Perioperative opioid dispensing and persistent use after benign hysterectomy: A systematic review and meta-analysis. Am. J. Obstet. Gynecol..

[10] Anyaehie K.B., Duryea E., Wang J., Echebelem C., Macias D., Sunna M., Ogunkua O., Joshi G.P., Gasanova I. (2022). Multimodal opioid-sparing pain management for emergent cesarean delivery under general anesthesia: A quality improvement project. BMC Anesthesiol..

[11] Choi J.B., Kang K., Song M.K., Seok S., Kim Y.H., Kim J.E. (2016). Pain characteristics after total laparoscopic hysterectomy. Int. J. Med. Sci..

[12] Nelson G., Bakkum-Gamez J., Kalogera E., Glaser G., Altman A., Meyer L.A., Taylor J.S., Iniesta M., Lasala J., Mena G. (2019). Guidelines for perioperative care in gynecologic/oncology: Enhanced recovery after surgery (eras) society recommendations-2019 update. Int. J. Gynecol. Cancer.

[13] Dong W., An B., Wang Y., Cui X., Gan J. (2021). Effect of multimodal analgesia on gynecological cancer patients after radical resection. Am. J. Transl. Res..

[14] Castro-Alves L.J., Oliveira de Medeiros A.C., Neves S.P., Carneiro de Albuquerque C.L., Modolo N.S., De Azevedo V.L., De Oliveira G.S. (2016). Perioperative duloxetine to improve postoperative recovery after abdominal hysterectomy: A prospective, randomized, double-blinded, placebo-controlled study. Anesth. Analg..

[15] Beattie W.S., Badner N.H., Choi P. (2001). Epidural analgesia reduces postoperative myocardial infarction: A meta-analysis. Anesth. Analg..

[16] Beattie W.S., Badner N.H., Choi P.T. (2003). Meta-analysis demonstrates statistically significant reduction in postoperative myocardial infarction with the use of thoracic epidural analgesia. Anesth. Analg..

[17] Popping D.M., Elia N., Marret E., Remy C., Tramer M.R. (2008). Protective effects of epidural analgesia on pulmonary complications after abdominal and thoracic surgery: A meta-analysis. Arch. Surg..

[18] Singh N., Sidawy A.N., Dezee K., Neville R.F., Weiswasser J., Arora S., Aidinian G., Abularrage C., Adams E., Khuri S. (2006). The effects of the type of anesthesia on outcomes of lower extremity infrainguinal bypass. J. Vasc. Surg..

[19] O’Neill A., Lirk P. (2022). Multimodal analgesia. Anesthesiol. Clin..

[20] Kharasch E.D., Avram M.J., Clark J.D. (2020). Rational perioperative opioid management in the era of the opioid crisis. Anesthesiology.

[21] Gaskin D.J., Richard P. (2012). The economic costs of pain in the united states. J. Pain..

[22] Larach D.B., Hah J.M., Brummett C.M. (2022). Perioperative opioids, the opioid crisis, and the anesthesiologist. Anesthesiology.

[23] Shanthanna H., Ladha K.S., Kehlet H., Joshi G.P. (2021). Perioperative opioid administration. Anesthesiology.

[24] Howard R., Kenney B., Brummett C., Waljee J., Englesbe M., Telem D. (2022). Prevalence and prescribers of preoperative opioid prescriptions in the us, 2008–2019. JAMA Netw. Open.

[25] Wong M., Vogell A., Wright K., Isaacson K., Loring M., Morris S. (2019). Opioid use after laparoscopic hysterectomy: Prescriptions, patient use, and a predictive calculator. Am. J. Obstet. Gynecol..

[26] Thompson J.C., Komesu Y.M., Qeadan F., Jeppson P.C., Cichowski S.B., Rogers R.G., Mazurie A.J., Nestsiarovich A., Lambert C.G., Dunivan G.C. (2018). Trends in patient procurement of postoperative opioids and route of hysterectomy in the united states from 2004 through 2014. Am. J. Obstet. Gynecol..

[27] Lirk P., Thiry J., Bonnet M.P., Joshi G.P., Bonnet F. (2019). Pain management after laparoscopic hysterectomy: Systematic review of literature and prospect recommendations. Reg. Anesth. Pain. Med..

[28] Kim C.H., Lefkowits C., Holschneider C., Bixel K., Pothuri B. (2020). Managing opioid use in the acute surgical setting: A society of gynecologic oncology clinical practice statement. Gynecol. Oncol..

